# Microneedle-based nanoporous gold electrochemical sensor for real-time catecholamine detection

**DOI:** 10.1007/s00604-022-05260-2

**Published:** 2022-04-07

**Authors:** Cristina Tortolini, Anthony E. G. Cass, Riccardo Pofi, Andrea Lenzi, Riccarda Antiochia

**Affiliations:** 1 Department of Chemistry and Drug Technologies, Sapienza University of Rome, P.le Aldo Moro 5, 00185 Rome, Italy; 2 Department of Chemistry & Institute of Biomedical Engineering, Imperial College, London, UK; 3 Department of Experimental Medicine, Sapienza University of Rome, Rome, Italy

**Keywords:** Microneedles, Nanoporous gold needle, Electrochemical sensor, Catecholamine, Dopamine, Epinephrine, Norepinephrine

## Abstract

Dopamine (DA), epinephrine (EP), and norepinephrine (NEP) are the main catecholamine of clinical interest, as they play crucial roles in the regulation of nervous and cardiovascular systems and are involved in some brain behaviors, such as stress, panic, anxiety, and depression. Therefore, there is an urgent need for a reliable sensing device able to provide their continuous monitoring in a minimally invasive manner. In this work, the first highly nanoporous gold (h-nPG) microneedle-based sensor is presented for continuous monitoring of catecholamine in interstitial fluid (ISF). The h-nPG microneedle-based gold electrode was prepared by a simple electrochemical self-templating method that involves two steps, gold electrodeposition and hydrogen bubbling at the electrode surface, realized by sweeping the potential between + 0.8 V and 0 V vs Ag/AgCl for 25 scans in a 10 mM HAuCl_4_ solution containing 2.5 M NH_4_Cl, and successively applying a fixed potential of − 2 V vs Ag/AgCl for 60 s. The resulting microneedle-based h-nPG sensor displays an interference-free total catecholamine detection expressed as NEP concentration, with a very low LOD of 100 nM, excellent sensitivity and stability, and fast response time (< 4 s). The performance of the h-nPG microneedle array sensor was successively assessed in artificial ISF and in a hydrogel skin model at typical physiological concentrations.

**Graphical abstract:**

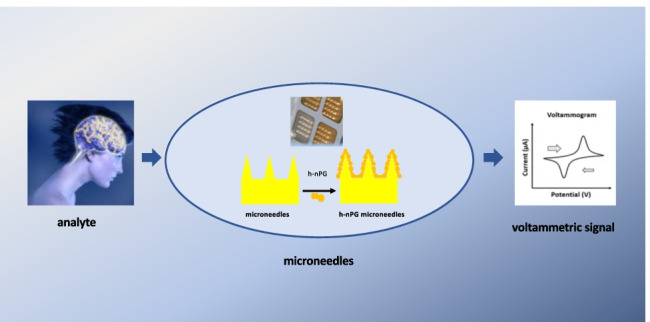

**Supplementary Information:**

The online version contains supplementary material available at 10.1007/s00604-022-05260-2.

## Introduction

Catecholamines (CAs) are monoamine compounds of the central and peripheral nervous system [1], which act as both neurotransmitters (NTs) and hormones. They include dopamine (DA), norepinephrine (NEP; also called noradrenaline (NA)), and epinephrine (EP; also called adrenaline (Adr)). They share the same catechol moiety, hence their name, and the same amino acidic precursor tyrosine. In resting individuals, endogenous CA concentrations in blood and interstitial fluid are approximately 150–800 ng/L for NEP and 10–50 ng/L for both NEP and DA [2]. The link between these amine compounds and human pathologies has been known for over 100 years [3]. Neurological disorders, such as Alzheimer and Parkinson’s diseases, the two most common neurodegenerative disorders; schizophrenia; and hyperactivity, are some examples of neurologic and psychiatric disorders related to catecholamine levels in the human body [3]. Normally, catecholamines and their metabolites are present in the body in small, fluctuating amounts, that only increase appreciably during and shortly after a stressful situation [4]. However, rare tumors called pheochromocytomas and neuroblastomas can produce large amounts of these hormones, resulting in increased concentrations in both blood and urine [5]. Therefore, there is a great need for a reliable sensing device to monitor catecholamine concentrations in biofluids, as they represent clinically relevant biomarkers for a particular disease or to monitor treatment efficacy. Moreover, the continuous real-time detection of CAs in both in vitro and in vivo experiments result of upmost importance in biomedical research to investigate the physiological role of these compounds in neurotransmissions events associated with various diseases or reward-related behaviors.

Considering that CAs have a very short half-live and are often secreted in an episodic manner, their levels might appear normal between secretory phases [6], creating a diagnostic problem. The opportunity for new methods, continuous monitoring, and real-time detection of CAs could overcome such issues, and offer a dramatic improvement in the diagnosis and follow-up management of diseases characterized by CA excess such as phaeochromocytomas and paragangliomas.

Numerous analytical methods have been developed including HPLC [7], UV spectrophotometry [8], mass spectroscopy [9], chemiluminescence [10], and electrochemical (EC) methods [2, 11]. In particular, EC methods have attracted a lot of attention, thanks to their favorable properties, such as simplicity, fast response time, high sensitivity, cost effectiveness, suitability for multiple detection, and possibility of miniaturization [2]. These features allow to overcome the most common drawbacks of the other techniques, such as time consuming and expensive laboratory equipment and trained personnel.

Electrochemical sensors and biosensors proved to be highly sensitive and selective techniques in the determination of CAs in biological fluids, while remaining inexpensive [2].

A critical problem associated with the detection of CAs is the coexistence of interfering compounds within the biological samples. The most important interfering compounds are ascorbic acid (AA) and uric acid (UA). They show similar oxidation potentials at conventional bare electrodes showing overlapping voltammetric signals, thus preventing a proper separation [12].

Another problem associated with the electrochemical detection of CAs is the so-called “biofouling process,” which is a passivation of the electrode surface, due the polymerization of the oxidation products of CAs [13].

To minimize interfering signals and to avoid surface passivation, several sensor surface modification approaches were undertaken. Initially, two strategies were adopted: covering of the electrode surface with conducting polymers [14, 15] and the formation of SAMs on oxide-free metals, especially gold surface. Unfortunately, the sensors based on these approaches require pre-concentration of CAs before measurements, with limited clinical application [2]. More recently, the coupling of nanotechnology with sensors/biosensors allowed the development of excellent models for both in vivo and in vitro quantitative analysis of CAs at physiological levels [15]. Metal nanoparticles [16], carbon nanotubes [17], and graphene [18] are the most used nanomaterials in the development of the new-generation EC sensors with enhanced performances, thanks to their high surface area, high electrocatalytic activity, improved selectivity, and fast mass transport [2]. Very recently, two-dimensional (2D) materials, such as transition metal dichalcogenides (TMDs), thanks to their tunable electronic/optical properties, are emerging as a new material platform to develop ultrasensitive sensors for CA detection [19]. Nanoporous metals [20, 21] have been also studied as sensitive electrodes thanks to their large surface area/volume ratio. In particular, nanoporous gold (nPG) has recently reached increasing attention because of its unique properties of high electrical conductivity, electrocatalytic activity, selectivity, and antifouling capacity [22–24].

However, most of the nano-based sensors allowed the determination of CAs either in plasma, with discomfort of the patient of having blood drawn; moreover usually blood needs to be taken after 30 min of supine rest with an in-dwelling catheter and, ideally, in the fasting state [25], or in 24-h urine samples [26].

Recent studies are investigating the possibility to measure CA concentrations in minimally invasive and more practical biofluids, such as sweat, thus allowing also a continuous monitoring with the development of wearable devices. At present, only two sensor platforms for the detection of DA in sweat have been reported, but unfortunately the detection limit is slightly higher than that obtained in plasma and urine [19, 27]. Moreover, these sensors cannot be as accurate as blood and urine tests because the content of sweat is more variable, being influenced by several factors.

Another interesting biological fluid for wearable sensors is the ISF, the fluid which surrounds the body cells, providing nutrients that directly diffuse from the capillary endothelium. Hence, many analytes, used in current clinical practice, are common to ISF and blood plasma, showing a reliable correlation with comparable concentration values [28] thanks to the equilibrium between the two fluids. Over the past three decades, researchers have utilized the ISF for non-invasive detection of metabolic diseases, organ failure, and drug efficacy, using the microneedle technology for ISF extraction and sensing [29]. More recently, the microneedle array technology is used for the assembling of wearable electrochemical sensors and biosensors [30]. Microneedle array-based sensors allow minimally invasive and continuous monitoring of important clinical biomarkers in ISF [28]. The method is well tolerated by patients, as the microneedles penetrate the stratum corneum at less than 1-mm depth, without reaching deep in the lower part of dermal compartment, where nerve and blood vessels are mainly present [31–34]. Moreover, microneedle-based devices have the advantage to accommodate multiple sensors on the same array, to provide multiplexed analysis.

**Scheme 1:**
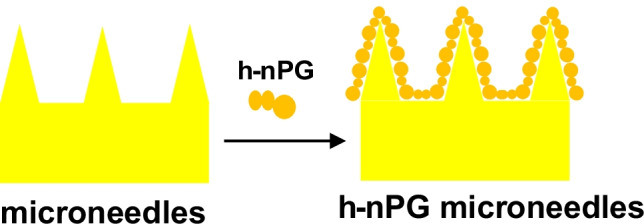
Schematic representation of the h-nPG-modified microneedle electrode

In this work, we describe the first example of a microneedle-based highly nanoporous gold (h-nPG) electrochemical sensor for selective and continuous monitoring of total CAs in ISF. The gold surface of the microneedles has been modified in a simple and scalable way, by a “self-templating” electrodeposition procedure of a h-nPG film, as shown in Scheme 1. The morphology was characterized through scanning electron microscopy (SEM) and electrochemical impedance spectroscopy (EIS). The microneedle-based h-nPG electrode showed significant selectivity for the detection of DA, NEP, and EP in presence of higher concentrations of ascorbic acid (AA) and uric acid (UA) and other interferents. The interesting analytical performances of the sensor were tested in artificial ISF and in a hydrogel skin model, showing promising results.

## Experimental

### Chemicals and reagents

Dopamine hydrochloride (DA), epinephrine bitartrate salt (EP), norepinephrine bitartrate salt monohydrate (NEP), sodium monobasic phosphate (Na_2_HPO_4_), sodium dibasic phosphate (NaH_2_PO_4_), potassium chloride (KCl), potassium ferricyanide (III) (K_3_[Fe(CN)_6_]), potassium ferrocyanide (II) (K_4_[Fe(CN)_6_]), gold (III) chloride solution, and ammonium chloride (NH_4_Cl) were purchased from Sigma-Aldrich (Buchs, Switzerland). All solutions were prepared in phosphate buffer 0.1 M, KCl 0.1 M, pH 7.4 (PBS). High-purity deionized water (resistance: 18.2 MΩ cm at 25 °C; TOC < 10 µg L^−1^) obtained from Millipore (Molsheim, France) has been used throughout experiments.

### Preparation of artificial interstitial fluid (ISF) and hydrogel skin model

The artificial interstitial fluid (ISF) was prepared by mixing 2.5 mMCaCl_2_, 5.5 mM glucose, 10 mM HEPES, 3.5 mM KCl, 0.7 mM MgSO_4_, 123 mM NaCl, 1.5 mM NaH_2_PO_4_, and 7.4 mM saccharose. The pH was adjusted to pH 7.4 [35].

The hydrogel skin model was prepared by dissolving 200 mg of agarose in 10 mL of 0.1 M PBS pH 7.4 and stirred at 120 °C in a glass until complete dissolution. Successively, the liquid is poured in a Petri dish (35 mm in diameter) containing 2.0 mL of artificial ISF and allowed to solidify. Finally, solutions containing proper NEP concentrations were drop-casted onto the Petri dish in order to have final NEP concentrations of 0, 5, 10, 20, 30, 50, 60, 75, 100, 185, 450, and 850 μM and allowed to diffuse for 1 h through the gel [34].

### Electrode preparation and modification

The microneedle-based h-nPG electrodes were modified by an self-templating electrodeposition method consisting of two steps: (i) sweeping the potential for 25 scans between + 0.8 V and 0 V vs Ag/AgCl at 50 mV s^−1^ in a 10 mM HAuCl_4_ solution containing 2.5 M NH_4_Cl and (ii) applying a fixed potential of − 2 V vs Ag/AgCl to the modified electrode for 60 s in the same solution, in order to allow the formation of pores, due to hydrogen bubbling. The H_2_ bubbles are generated in situ in a solution containing H^+^ (self-templating procedure, without the use of an external template). Finally, the electrodes were further activated in 0.5 M H_2_SO_4_, by running CVs between 0 and + 1.7 V vs Ag/AgCl at a scan rate of 100 mV s^−1^ for 25 cycles, until a well-defined CV was obtained [36].

### SEM experiments

Scanning electron microscopy (SEM) measurements were carried out with a High-Resolution Field Emission Scanning Electron Microscopy (SEM) (HR FESEM; Zeiss Auriga Microscopy, Jena, Germany), in order to investigate the morphology of the bare and modified screen-printed electrodes (SPEs).

Energy-dispersive X-ray spectroscopy (XPS) measurements were performed in order to evaluate the Au content of the h-nPG-modified screen-printed electrode. The EDX spectrum and data were collected during sample surface scanning by SEM electron probe.

### Electrochemical measurements

All electrochemical measurements were performed in a 10-mL thermostated glass cell (model 6.1415.150, Metrohm (Herisau, Switzerland)) with a conventional three-electrode configuration with an Ag/AgCl/KCl_sat_ (198 mV vs NHE) as reference electrode (cat. 6.0726.100, Metrohm, Herisau, Switzerland), a glassy carbon rod as a counter electrode (cat. 132 6.1248.040, Metrohm, Herisau, Switzerland), and a gold classical electrode (diameter 2 mm) as working electrode (Au, cat. 6.1204.320, Metrohm, Herisau, Switzerland). A gold screen-printed electrode (Au-SPE, 220BT Metrohm, Herisau, Switzerland, Aux: gold; Ref: silver, diameter 4 mm) was also used as working electrode.

The microneedle-array Au microneedle electrodes were fabricated at Glasgow University [32] and metallized by Torr Scientific Ltd. (Bexhill). They are based on a polycarbonate scaffold (0.5 × 0.5 × 0.02 cm) with 64 microneedles divided as four 4 × 4 arrays. Each pyramid showed the following dimensions: base 0.06 cm, height 0.1 cm, and 4 × 4 array area 0.2 cm^2^. On this platform, three electrodes were used as working electrodes (gold), while the fourth one was the reference electrode (silver). In our work, we used the gold array as working electrode and external Ag/AgCl/KCl_sat_ and glassy carbon rod (described above) as reference and counter electrode, respectively.

EIS experiments were carried out at equilibrium potential called open-circuit potential (OCP) without potential of the redox probe (0.22 V vs Ag/AgCl), bias voltage in the frequency range of 0.1–10^3^ Hz using an AC signal of 10-mV amplitude at a formal potential of the redox probe (0.22 V vs Ag/AgCl), using Autolab Potentiostat/Galvanostat (Eco Chemie, Netherlands). EIS measurements were performed using 10 mL of PBS solution containing mixture of 5 mM Fe(CN)_6_^3−^/Fe(CN)_6_^4−^, as electrochemical probe.

## Results and discussion

### SEM characterization and microanalysis of h-nPG microneedle electrodes

**Fig. 1:**
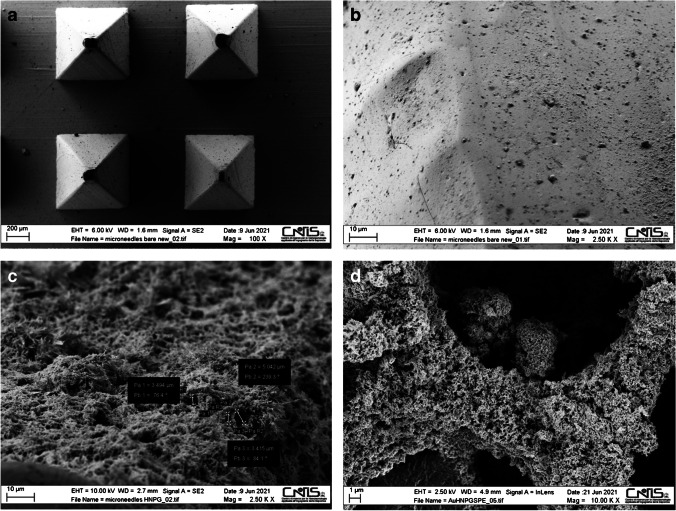
SEM images of Au bare microneedles (**A** and **B**) and h-nPG microneedle electrode (**C** and **D**)

The morphology and components of the microneedles were characterized by SEM and SEM–EDX experiments, respectively. SEM images were used to investigate the physical appearance and the surface characteristics of the microneedle-based electrode before and after the electrodeposition of the h-nPG film. Figure 1 shows the SEM images of the bare (panels A and B) and the h-nPG microneedle-based electrodes (panels C and D), at different magnifications. It is possible to note that a h-nPG film is deposited onto the microneedle-based electrode surface after the electrodeposition method, exhibiting evenly distributed nanopores with diameter of a few nanometers (panel C), typical of a sponge-like structure (panel D).

The EDX microanalysis was carried out in order to exclude any potential contaminant in the electrodeposited film. The EDX spectrum and the content percentage of each element are shown in Fig. S1, panels A and B. The spectrum clearly indicates that the product is mainly composed of Au with traces of C, O, and N. It depicts a strong band at 2.15 keV, which is the typical optical adsorption of gold nanocrystals. The high- and low-intensity bands at 0.25 and 9.71 keV also correspond to Au, in accordance with the EDX energy table. It is interesting to note the absence of chloride as possible contaminant, although the electrodeposited film is generated from a gold chloride solution.

### Electrochemical behavior of h-nPG microneedle electrodes

The electrochemical behavior of the h-nPG microneedle electrodes was monitored by cyclic voltammetry (CV) and electrochemical impedance spectroscopy (EIS) experiments.

### CV characterization

The electrochemical characterization of the microneedle electrode before and after h-nPG electrodeposition has already been investigated by CV in a solution of Fe(CN)_6_^3−^/^4−^, as reported in our previous work [36]. It is interesting to note that the enhancement of both anodic and cathodic peak current densities after the h-nPG electrodeposition was much larger than that registered with planar gold electrodes after the same modification. The increase of the electroactive surface area after the formation of the h-nPG film obviously occurs in both cases, but the particular geometry of the microneedles seems to play an important role, allowing a better electrodeposition of the h-nPG film and therefore achieving a larger electroactive surface area [36].

### EIS characterization

**Fig. 2:**
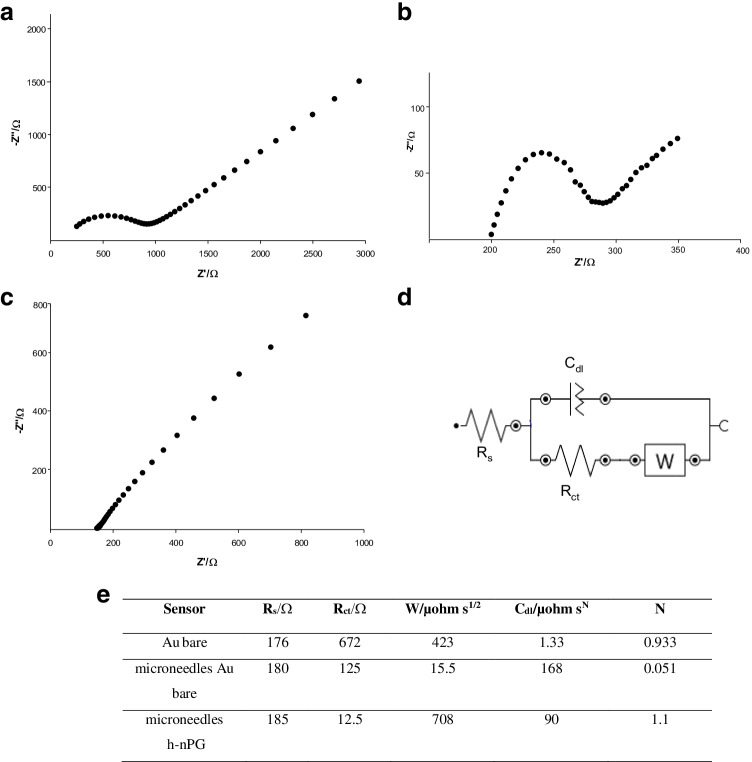
The Nyquist plots of bare Au electrode (**A**), microneedle bare Au electrode (**B**), and microneedle h-nPG electrode (**C**) in 5 mM [Fe(CN)_6_]^3−/4−^  + 0.1 M KCl and the equivalent circuit used for fitting the experimental data (**D**). (E) The EIS results from the electrochemical Nyquist plot fitting

EIS was carried out to investigate the impedance alteration at the electrode-solution interface after the h-nPG modification of the microneedle-based gold electrode. It is known that the Nyquist plot consists of a semicircular part and a linear part. At high frequencies, the semicircle part corresponds to the electron transfer resistance (*R*_ct_), measured as the semicircle diameter, which gives information about the electron transfer kinetics on the electrode surface. At low frequencies, the linear part is indicative of systems under diffusion-controlled current [37]. Figure 2 (panels A–C) are the Nyquist plot of unmodified Au (A) and microneedle Au electrodes before (B) and after (C) the modification with the h-nPG film. It is possible to observe a clear reduction of the semi-circular part when the microneedle-based electrode is used compared to Au bare electrode. This can be ascribed to a better conductivity, probably obtained thanks to the particular geometry of the microneedles [36]. A further decrease of the charge-transfer resistance is observed with the h-nPG-modified microneedle electrode, indicating the good electrical conductivity of the nanoporous film, which promotes a higher electron transfer rate in the redox probe.

The EIS data were successively fitted, and the corresponding equivalent circuit is reported in Fig. 2, panel D. It is interesting to note, as reported in Fig. 2 panel E, that there is a drastic decrease of the *R*_ct_ value with h-nPG microneedle electrode (1 10^−6^ Ω), compared to Au bare microneedle electrode (125 Ω), suggesting that the nanoporous structure of the h-nPG film allows a higher electron transfer rate in the redox probe, thanks to the high conductivity of the nanostructures.

### Electrocatalytic oxidation of DA, EP, and NEP at h-nPG microneedle electrode

**Fig. 3:**
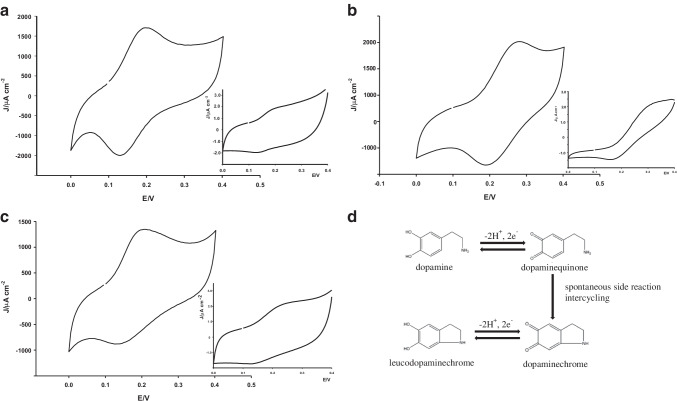
CVs of 0.1 mM DA (**A**), EP (**B**), and NEP (**C**) in PBS 0.1 M, KCl 0.1 M, pH 7.4 at 25 mV s^−1^ using h-nPG microneedle electrode. In the insets Au bare microneedle electrode. (D) A possible DA oxidative pathway

The electrocatalytic behavior of DA, EP, and NEP at bare and modified microneedle electrode was investigated in PBS pH 7.4 containing 100 μM of each catecholamine, as shown in Fig. 3 (panels A, B, and C). It is clearly noted that microneedle-modified electrodes showed significantly higher DA, EP, and NEP oxidation currents (1000-fold) and lower oxidation potentials, compared to bare microneedle electrodes. The large oxidation currents and low oxidation potential values shown by the h-nPG microneedle electrodes suggest the enhanced electrocatalytic behavior of the nanoporous film toward oxidation of each catecholamine, compared to bare microneedle electrodes. Moreover, the modified electrodes showed sharper and well-defined oxidation peaks compared to bare electrodes. This result might be due to the faster electron transfer rate at the modified electrode/solution interface, thanks to the increased electrical conducting surface area of the nanoporous film and the entrapment of the electroactive species inside the nanoporous, which allows negligible mass transfer and shorter diffusion times [38, 39].

CV of bare microneedle electrodes show a total electrochemically irreversible behavior towards detection of NEP, as no defined anodic and cathodic peak currents were observed (Fig. 3, inset panel C), and a quasi-reversible behavior in the case of DA and EP, with a potential peak separation (Δ*E*_*p*_) of about 65 mV vs Ag/AgCl (Fig. 3, inset panels A,B).

The combined effect of the improved peak current responses and the low Δ*E*_*p*_ obtained with h-nPG microneedle electrode for DA, EP, and NEP must be due to the synergistic effect of the greater active surface area of the h-nPG film compared to bare electrode, as well as the faster electron transfer rate. The reversible behavior observed with all CAs after electrode modification provides clear evidence of the catalytic effect of the nanoporous film, which act as a promoter to enhance the electron transfer rate of the electrochemical reaction. The heterogeneous electron transfer rate constants (k^0^cm s^−1^) for DA, EP, and NEP by h-NPG microneedle electrode have been calculated with a method proposed by Lavagnini et al. that merges the Klingler–Kochi and Nicholson and Shain methods developed for irreversible and reversible systems, respectively [40, 41] and resulted to be 3.5 × 10^−3^, 4.0 ±  × 10^−3^, and 4.2 × 10^−3^ cm s^−1^, respectively.

It is reported in literature that the oxidation of DA, EP, and NEP occur via a complex two-electron two-proton transfer process, with parallel spontaneous side reactions leading to the formation of different oxidized species, which may inhibit the electron transfer, thus decreasing the electrode response sensitivity [42, 43]. The mechanism of the oxidation/reduction process of DA on the h-nPG electrode has been investigated by recording a cyclic voltammogram in a larger potential window, from − 0.4 to 0.4 V vs Ag/AgCl. It is interesting to note that the h-nPG microneedle electrode shows one sharp oxidation peak in the positive scan from 0 to 0.4 V and two well-defined cathodic peaks at about 0.16 and − 0.2 V vs Ag/AgCl in the reverse scan, as shown in Fig. S2 (panel A). The well-resolved cathodic peaks can be ascribed to the subsequent reduction of two oxidized species: dopaminequinone to dopamine and dopaminechrome, formed via a spontaneous intramolecular cyclization of dopaminequinone, to leucodopaminechrome, as schematized in Fig. 3, panel D [44]. In the potential range from − 0.4 to 0 V a second small oxidation peak occurs, as a counterpart of the cathodic peak at − 0.2 V, corresponding to the oxidation of leucodopaminechrome to dopaminechrome. Similar steps can be assumed for EP and NEP oxidation/reduction mechanisms (Fig. S2, panel B), due to the similarities of their reverse CV scans with DA (curves not shown).

### Effect of scan rate

**Fig. 4:**
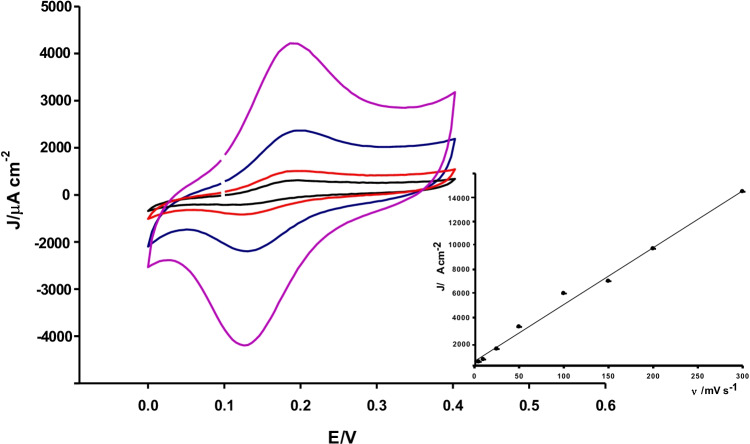
CVs of 0.1 mM DA, with microneedle h-nPG electrode at the following scan rates: 5 (black), 10 (red), 25 (blue), and 50 mV s^−1^ (pink) in 0.1 M PBS and 0.1 M KCl (pH 7.4). In the inset, the plot of the peak current density vs scan rate (5–300 mV s^−1^) (*n* = 3)

The scan rate effect on the anodic peak currents of DA, EP, and NEP was investigated at the developed nanoporous microneedle electrode at a fixed concentration (100 μM) of each catecholamine. CVs of DA at different scan rates (5–300 mV s^−1^) have been registered and in Fig. 4 are reported between 5 and 50 mV s^−1^. The anodic peak current increases linearly with the scan rate with the following regression equation: *J*_*pa*_/mA cm^−2^ = 0.04 ν/mV s^−1^ + 1.9 (*R* = 0.999) (Table S1). The linear dependence of *J*_*pa*_ versus scan rate indicates a typical surface adsorption behavior (insets Fig. 4, panel A) [45], as already reported for different gold modified electrodes [46]. The adsorption of DA on the surface of h-nPG electrode can be ascribed to a possible interaction between the amino group of DA and the nanoporous gold surface [47].

Different results were obtained for EP and NEP. The *J*_*pa*_ values of both EP and NEP resulted to be linear with *ν* at low scan rates, in the range 5–100 mV s^−1^, suggesting a surface-controlled process. At higher scan rates (between 150 and 300 mV s^−1^), the plot of *J*_*pa*_ versus the square root of scan rate resulted to be linear, suggesting a diffusion rather than a surface-controlled process (Fig. S3, panels A and B). This particular trend may be attributed to a mixed diffusion-adsorption control of the redox process [38]. The linear regression equations of *J*_*pa*_ versus *ν* and *ν*^1/2^ for EP and NEP are reported in Table S1. It is interesting to note that this particular behavior is not obtained for EP and NEP with other nanocomposite-modified electrodes, showing a diffusion-controlled process [48]. Therefore, the surface phenomena reported for the developed nanoporous microneedle electrodes could be ascribed to their particular structure.

In addition, the charge transfer coefficient α and the standard heterogeneous rate constant (*k*_*s*_) were calculated according to Laviron equation [45], by using the variation of the anodic and cathodic peak potentials versus logarithm of scan rate and the Laviron equation, respectively. At higher scan rates, the dependence of *E*_*p*_ versus log *ν* is linear (figure not shown) and the slopes of the linear part are − 2.303 *RT*/ α*nF* and 2.303 *RT*/(1 −  α) *nF* for cathodic and anodic peaks, respectively [45]. The charge transfer coefficients (α) calculated as the mean of the cathodic and anodic values resulted to be 0.69 for DA, 0.96 for EP, and 0.41 for NEP, respectively.

From the values of Δ*E*_*p*_ corresponding to different scan rates, average values of *k*_*s*_ were found to be 0.01, 0.002, and 0.003 s^−1^ for DA, EP, and NEP, respectively.

The surface coverages (Γ) of DA, EP, and NEP onto the microneedle-modified electrode were calculated from the plot of *I*_*p*_ versus scan rate ν, according to Eq. (1) [45]:

1 $${i}_{p}={n}^{2}{F}^{2}vA\Gamma /4RT$$

where *n* is the number of electrons; *F*, *R*, and *T* are the Faraday constant, the molar gas constant, and the absolute temperature; *ν* is the scan rate; and Γ is the surface coverage. Assuming *n* = 2 for the three compounds, the Γ values of DA, EP, and NEP were found to be 5.35 × 10^−8^, 0.06 × 10^−8^, and 0.07 × 10^−8^ mol cm^−2^, respectively. The data showed a similar order of magnitude compared to Γ values calculated for CAs with different electrode nanomodification reported in literature [46], indicating an increased adsorptivity of the h-nPG-nanomodified microneedle electrode.

The Tafel values were calculated by using Eq. (2), by plotting *E*_*p*_ versus log ν (Fig. S4):

2 $${E}_{p}=\left(b/2\right)logv+k$$

where *E*_*p*_ is the anodic peak potential (V), ν is the scan rate (mV s^−1^), and *b* represents the Tafel value. The obtained values for the microneedle-modified electrode, in a 0.1 mM solution of each catecholamine, were 164, 74, and 80 mV dec^−1^ for DA, EP, and NEP, respectively. A high Tafel value indicates a larger analyte adsorption. The Tafel values were found in the order DA > NEP > EP, confirming the larger adsorption of DA on the highly porous electrode surface.

The logarithm of the peak current (log *i*_*p*_) vs logarithm of the scan rate (log ν) for DA, EP, and NEP is reported in Fig. S5. The slope values resulted to be 0.55, 0.20, and 0.36 for DA, EP, and NEP, respectively. It is known that a log ν value equal to 0.5 is typical of a diffusion-controlled process; a higher value is indicative of an adsorption process and a lower value of a mixed diffusion-adsorption process. Therefore, the results confirm that DA showed an adsorption-controlled process, on the contrary both EP and NEP a mixed diffusion-adsorbed process.

#### Apparent diffusion coefficients of DA, EP, and NEP at h-nPG microneedle electrode

The study of the dependence of the anodic peak currents against the scan rate was used for the estimation of the “apparent” diffusion coefficients *D*_app_ for DA, EP, and NEP at h-nPG microneedle electrodes. The *D*_app_ values were calculated from the slope of *i*_*p*_ vs ν^1/2^ plots, using the Randles–Sevcik equation:

$${i}_{p}=2.69\times {10}^{5}{n}^{3/2} A {C}^{0} {D}^{1/2} {v}^{1/2}$$

where *i*_*p*_ is the peak current density (A cm^−2^), *n* is the number of electrons, *A* is the geometrical microelectrode area (cm^2^), *C*_0_ is the analyte concentration (5 × 10^−6^ mol cm^−3^), ν is the scan rate, and *D* is the diffusion coefficient of the electroactive species (cm^2^ s^−1^).

The *D*_app_ value obtained for DA, EP, and NEP resulted to be 5.45 × 10^−6^, 4.5 × 10^−6^, and 7.3 × 10^−6^ cm^2^ s^−1^, respectively, consistent with values reported in literature [47].

### Selectivity

**Fig. 5:**
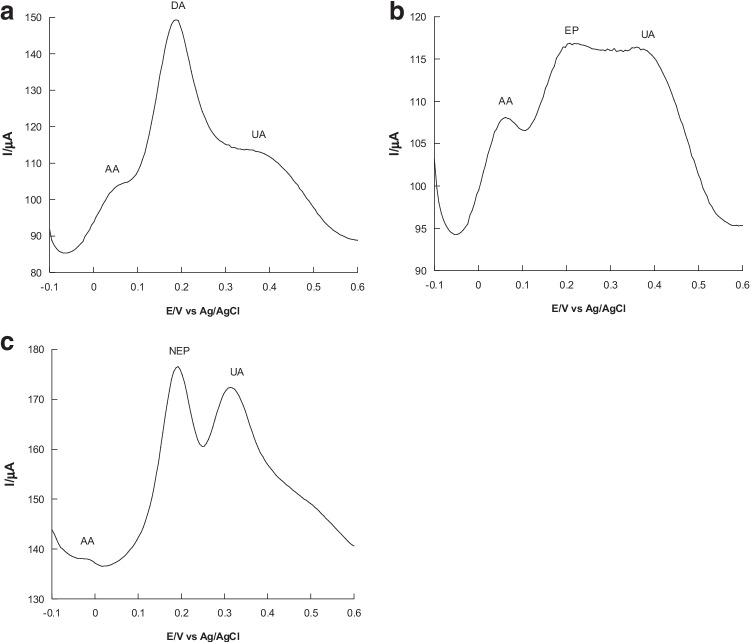
DPVs of 0.5 mM DA (**A**), EP (**B**), and NEP (**C**) 0.5 mM in presence of 4 mM AA and 0.6 mM UA with h-nPG microneedle electrode

Selectivity of the h-nPG microneedle electrode for DA, EP, and NEP was investigated separately by recording sensor responses in exposure to other potential interferents, such as AA and UA, which most frequently coexist in biological matrices. It is fundamental to selectively detect each single catecholamine in the presence of AA and UA, as they usually show overlapping voltammetric curves at both unmodified and chemically modified electrodes. Figure 5 shows the differential pulse voltammograms of ternary mixtures of a single catecholamine (DA, EP, or NEP) and AA and UA. It is possible to note three sharp, well-resolved anodic peaks at about 60, 200, and 360 mV for AA, each catecholamine and UA. Thus, the separations of the peaks were large enough to determine DA, EP, and NEP individually in the presence of a similar concentration of UA and a ten times higher concentration of AA.

Successively, other possible interfering compounds have been tested by examining the DPV responses of 200 μM of DA, EP, and NEP in the presence of various interferents, such as L-cysteine, L-lysine, urea, citric acid, NaCl, and folic acid at 500 μM and glucose at 5 mM. The results are shown in Table 1. No significant influence was observed for all interfering compounds tested with maximum percentages of signal change of 4% for DA, 3% for EP, and 2% for NEP.

**Table 1:** Influence of interfering compounds on DA, EP, and NEP response (200 μM) of the h-nPG microneedle sensor

Interfering compound	DA (μM)	Recovery (%)	EP (μM)	Recovery (%)	NEP (μM)	Recovery (%)
Glucose	197	98.5	198	99	196.5	98.2
L-cysteine	195	97.5	196	98	198	99
L-lysine	194	97	195	97.5	197	98.5
Urea	201	100.5	196	98	198	99
Citric acid	192	96	194	97	196	98
NaCl	196	98	203	101.5	201	100.5
Folic acid	204	102	195	97.5	198	99

Interference amount added = 500 μM; glucose added = 5 mM

### Reproducibility, repeatability, and stability

The reproducibility of the catecholamine sensors was evaluated using five independent h-nPG microneedle electrodes by measuring the peak currents of 10 µM of each catecholamine. The RSD of DA, EP, and NEP resulted to be 3.08%, 3.64%, and 2.24%, respectively, suggesting the excellent reproducibility of the sensors which attests that the h-nPG microneedle electrodes do not undergo significant surface biofouling during measurements.

In order to investigate the repeatability, the three sensors were used to perform 10 repetitive measurements in PBS containing 10 µM of each catecholamine. The RSD resulted to be 2.08%, 2.79%, and 1.99% for DA, EP, and NEP, respectively, showing remarkable repeatability.

The stability and lifetime of the sensors were investigated by testing the DPV responses of the three sensors for 10 measurements every day over a period of 30 days, for a 10 µM solution of each catecholamine, as reported in Fig. S6 During this period, the sensors were stored dry in a refrigerator (4 °C). The DPV peak currents of DA, EP, and NEP remain 91.8%, 92.6%, and 93.4% of the original signals, respectively, showing a slow progressive current decrease. The excellent stability is probably derived from a synergic effect of the nanostructuration and the particular geometry of the microneedle-based electrode, which avoids electrode biofouling. This is a fundamental requirement for a sensor for transdermal continuous analysis.

### h-nPG microneedle total catecholamine sensors

**Fig. 6:**
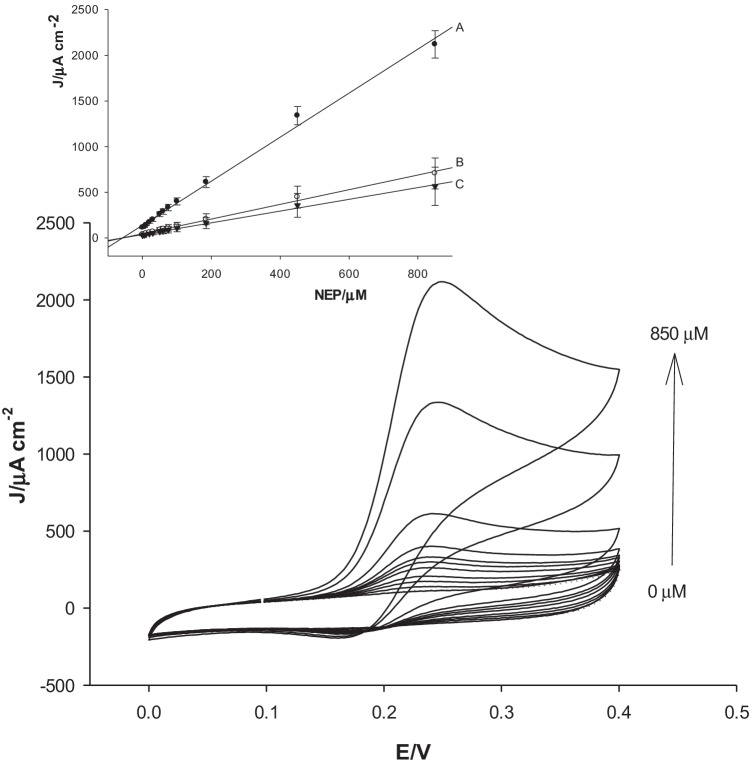
CVs of microneedle h-nPG sensor in 0.1 M PBS, pH = 7.4, and KCl = 0.1 M, at the following NEP concentrations: 0, 5, 10, 20, 30, 50, 60, 75, 100, 185, 450, and 850 μM. Experimental conditions: scan rate = 25 mV s^−1^; *T* = 25 °C. In the inset: calibration curve of NEP in phosphate buffer (**A**), in artificial ISF (**B**), and in gel skin model (**C**) (*n* = 3)

The total catecholamine concentration is expressed as NEP concentration, thanks to the best selectivity shown by the NEP sensor, in order to minimize interferences. The influence of the concentration of NEP at the h-nPG microneedle electrode was studied using cyclic voltammetry experiments. CV response for NEP is investigated at various spiked NEP concentrations varying from 1 to 850 μM. The peak current increased with increasing NEP concentration, as shown in Fig. 6. The calibration curve was plotted between the oxidation peak currents (more visible than cathodic peaks) and NEP concentration, as reported in the inset of Fig. 6 (curve A), displaying a good linearity in the above concentration range. The sensitivity and detection limit were calculated from the plot and found to be 2.4 ± 0.05 μA μM^−1^ cm^−2^ and 0.1 μM at *S*/*N* = 3, respectively.

Table 2 shows a comparison with the results reported in literature for various nanostructured modified sensors for CA detection. Different nanomaterial-based sensor platforms have been constructed, thanks to the excellent conductivity, biological compatibility, and also enzyme-mimicking ability of the nanomaterials. In particular, three nanoporous modified (no microneedle-based) sensors are reported in the upper part of Table 2. The h-nPG microneedle sensor proposed in this study shows a lower detection limit and a wider linear range compared to the other nanoporous gold no microneedle-based sensors published in literature. Moreover, by comparing the results of the proposed sensor with no microneedle-based sensors modified with different nanomaterials (lower part of Table 2), it is possible to observe a generally wider linear range, with a LOD value higher in some cases [49–52].

**Table 2:** Comparison of analytical parameters of nanostructured sensors reported in literature for CA detection

Electrode	Modification strategy	CA	Linear range (µM)	LOD (µM)	Ref
nPG/GCE	Dealloying	DA	-	0.2	[48]
nPG/AuE	Electrodeposition	DA	0.1–40	-	[39]
nPG/AuE	Electrodeposition	EP	50–1000	1.8	[53]
h-nPG/Au microneedles	Electrodeposition	NEP	5–850	0.1	This work
N-UNCD/TiE	Microwave plasma CVD	DA	1–30	-	[59]
ECR/GCE	Electrodeposition	NEP	2–50	1.5	[60]
TiO_2_-BH/CPE	Drop casting	NEP	4–1100	0.5	[49]
NMM/CPE	Mixing NMM with graphite powder/paraffin oil into a paste	NEP	0.07–2000	0.04	[61]
Tyr/CDs/CA/AuE	Cross-linking/physical adsorption	NEP	1–200	0.2	[50]
CeO_2_-PEDOT/MWCNT/GCE	Electrodeposition/drop-casting	DA	0.1–10 40–400	0.03	[51]
CNT-Nb	Physical vapor deposition	DA	-	0.011	[52]
RuS_2_NPs/GCE	Hydrothermal synthesis	DA	0.1–10	0.074	[54]

In the upper part, nanoporous gold-modified sensors

*nPG* nanoporous gold, *GCE* glassy carbon electrode, *AuE* gold electrode, *N-UNCD* nitrogen-incorporated ultrananocrystalline diamond, *TiE* titanium electrode, *CVD* chemical vapor deposition, *ECR* eriochrome cyanine, *CPE* carbon paste electrode, *BH* = *2,2-*[1, 2] buthanediylbis(nitriloethylidyne)]-bis-hydroquinone, *NMM* nanostructured mesoporous material, *Tyr* tyrosine, *CDs* carbon dots, *CA* cysteamine, *Ce* = *2-PEDOT* nanoceria-poly (3,4-ethylenedioxythiophene, *CNT-Nb* carbon nanotube-coated niobium, *RuS*_*2*_*NPs* ruthenium disulfide nanoparticles

### Application of the h-nPG microneedle NEP sensor in artificial ISF and in a gel skin model

Continuous monitoring of CA levels is a significant task in medical diagnostics. Therefore, the analytical performances of the h-nPG microneedle sensor for NEP detection were further assessed in artificial ISF and in a gel skin model, both spiked with NEP. The skin model consists of an agarose (2%) hydrogel opportunely embedded in artificial ISF, in order to mimic the human dermis. The microneedle sensor is immersed into the artificial ISF in the first case and inside the gel in the latter. The calibration curves obtained from the CV responses at different NEP concentrations in artificial ISF and in the skin model are shown in the inset of Fig. 6 (curves B, C). Compared to the results obtained with the same sensor in PBS, the linear ranges are the same, while the sensitivity values slightly lower, resulting 0.80 ± 0.02 μA μM^−1^ cm^−2^ and 0.64 ± 0.01 μA μM^−1^ with corresponding LOD values of 0.25 μM and 0.3 μM, for ISF and gel skin model, respectively. This result can be ascribed to the biofouling effect due to protein constituents of the ISF, which causes a slight decrease of the analytical signal.

## Conclusions

Herein, we developed the first example of an electrochemical sensing platform for minimally invasive and continuous monitoring of total catecholamine, based on a h-nPG microneedle electrode.

The electroanalytical characterization of the proposed platform indicates the existence of a high electrocatalytic activity in the h-nPG film and its good stability. The self-templated electrochemical deposition resulted to be a fast, reproducible, and eco-friendly technique of electrode modification that avoids the harsh chemicals required for chemical dealloying.

The resulting h-nPG microneedle sensor displays attractive analytical performances for DA, EP, and NEP detection with high sensitivity and selectivity, good reproducibility, and excellent stability. Importantly, it is perfectly biocompatible and allows real-time monitoring of DA, EP, and NEP.

Moreover, the proposed sensor was tested for “in vitro” measurements in artificial ISF and in a gel skin model, maintaining high sensitivity, a wide linear dynamic range, and high selectivity, compared to PBS.

However, differentiation between the current responses achieved for the three catecholamines was not possible, due to their similar structures giving overlapping oxidation signals.

Future studies will be required to better investigate the correlation between CA concentration in blood and in ISF. It is known that ISF contains similar information to plasma with most small uncharged analytes present at near-equal concentrations in both biofluids, such as glucose and cortisol, as they freely interchange between them by diffusion through the plasma membrane. However, some charged analytes, such as CAs, are commonly found in plasma or in ISF at slightly different concentrations [54], as they cannot traverse the plasma membrane by a simple diffusion process but are shuttled in one of the two compartments [28].

A future goal will be the biouse of the proposed electrochemical platform to develop wearable sensors able to detect CA threshold values to provide diagnostic tools for pathological conditions associated with CA excess (pheochromocytomas and paragangliomas), and thereafter to correlate neurological conditions, such as schizophrenia or Parkinson’s to fluctuations in catecholamine levels.

Further efforts will focus on the integration of the microneedle sensor with a wireless electronic interface and wireless transmission for decentralized health status assessment and treatment [55–58].

Although the great potential of the proposed CA sensors in terms of real-time, low cost, high sensitivity, and selectivity, further research and investigations are required along with in vivo studies before practical applications are considered.

## Supplementary Information

Below is the link to the electronic supplementary material.

Supplementary file1 (DOCX 514 KB)
