# Supplementary material for: Microneedle-based nanoporous gold electrochemical sensor for real-time catecholamine detection
Source: Mikrochim Acta. 2022 Apr 7;189(5):180. doi: 10.1007/s00604-022-05260-2 (PMC8989844; doi:10.1007/s00604-022-05260-2)
Supplement: Supplementary file 1 — Supplementary file1 (DOCX 514 KB) [file 604_2022_5260_MOESM1_ESM.docx]

**Electronic Supplementary Material**

**Microneedles-based nanoporous gold electrochemical sensor for real time catecholamine detection**

**Cristina Tortolini^a^, Anthony E.G. Cass^b^, Riccardo Pofi^c^, Andrea Lenzi^c^, Riccarda Antiochia^a,*^**

*^a^Department of Chemistry and Drug Technologies, Sapienza University of Rome, P.le Aldo Moro 5, 00185, Rome, Italy*

*^b^Department of Chemistry & Institute of Biomedical Engineering, Imperial College, London, UK*

^a^*Department of Experimental Medicine, Sapienza University of Rome, Rome, Italy*

* Corresponding author.

*E-mail address*: riccarda.antiochia@uniroma1.it

(A)

(B)

Spectrum: Microneedles_ 1

Element Series unn. C norm. C Atom. C Error (1 Sigma)

[wt.%] [wt.%] [at.%] [wt.%]

--------------------------------------------------------

Gold M-series 86.98 91.24 40.08 3.45

Carbon K-series 6.23 6.53 47.05 1.16

Oxygen K-series 1.12 1.17 6.34 0.33

Nitrogen K-series 1.01 1.06 6.53 0.38

--------------------------------------------------------

Total: 95.33 100.00 100.00

**Fig. S1** EDX spectra of h-nPG microneedles electrode (panel A) and chemical composition (%) of h-nPG microneedles electrode (panel B) obtained from EDX analysis.

(A)

(B)


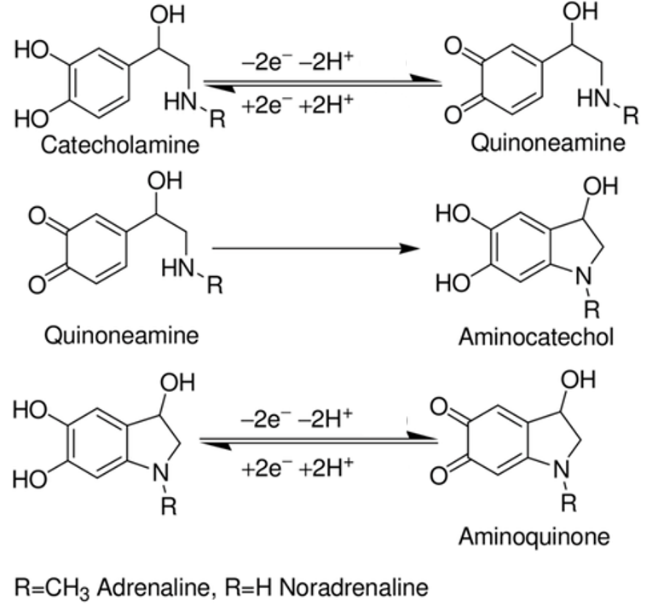

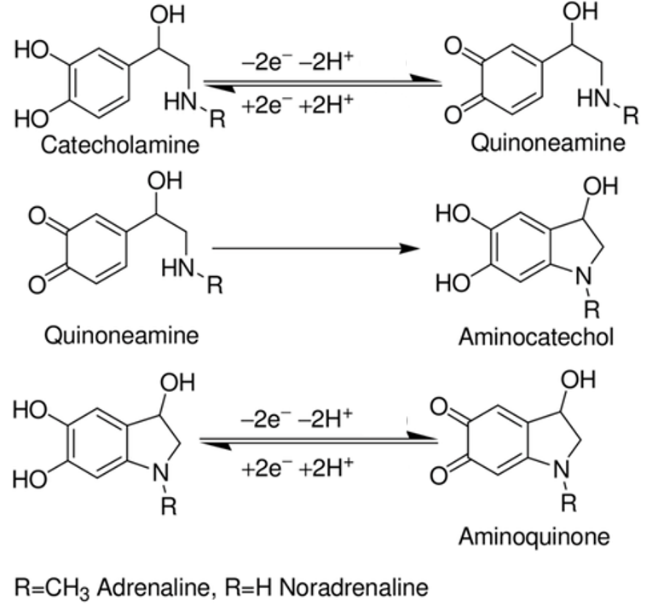


**Fig. S2** CV of 0.1 mM DA with h-nPG Au classical electrode at 25 mV s^-1^ (panel A); Possible EP and NEP oxidative pathways (panel B).

(A)

(B)

**Fig. S3** CVs of 0.1 mM EP (A) and NEP (B) with microneedles h-nPG electrode at different scan rates (5-50 mV s^-1^), in 0.1 M PBS, 0.1 M KCl (pH 7.4). In the insets, the plot of the peak current density vs. scan rate and/or square root of scan rate (5÷300 mV s^-1^).

**Fig. S4** Plots of peak potential E_p_ vs logν of h-nPG microneedles electrode in PBS buffer, containing 1.0 mM of DA (○), EP (▼) and NEP (●).

**Fig. S5** Log I_p_ vs. log v plots of the three catecholamines: DA (□), EP (●) and NEP (○), respectively.

(A)

(B)

(C)

**Fig. S6** Stability measurements over a period of 30 days in presence of 10 μM of DA (A), EP (B) and NEP (C) in 0.1 M PBS, 0.1 M KCl (pH 7.4), (n=10).

**Table S1**. Linear regression equations of J_pa_ versus ν and ν^1/2^ for DA, EP and NEP.

| **Catecholamine** | **Linear regression equation** | |
| --- | --- | --- |
|  | **ν / 5-100 mV s^-1^** | **ν / 150-300 mV s^-1^** |
| **DA** | J_pa_/mA cm^-2^ = 0.04 ν/mVs^-1^ + 1.9, R = 0.999 | |
| **EP** | J_pa_/ mA cm^-2^ = 0.04 ν/mVs^-1^ + 0.7, R = 0.992 | J_pa_/mA cm^-2^ = 1.2 ν^1/2^/(mVs^-1^)^1/2^ – 7.8, R = 0.999 |
| **NEP** | J_pa_/mA cm^-2^ = 0.02 ν/mVs^-1^ + 0.8, R = 0.994 | J_pa_/mA cm^-2^ = 0.6 ν^1/2^/(mVs^-1^)^1/2^ – 3.8, R = 0.998 |
